# The Use of Lime Vapor in Membranous Croup

**Published:** 1873-08-15

**Authors:** John Bartlett


### THE USE OF LIME VAPOR IN MEMBRANOUS CROUP.

SUBSTANCE OF A PAPER READ BY JOHN BARTLETT, M.D., BEFORE THE CHICAGO SOCIETY OF PHYSICIANS AND SURGEONS, AT THE MEETING HELD MAY 26TH, 1873.

Successes in the treatment of membranous croup with the various form of lime inhalations have been reported sufficiently often to direct the attention of the profession to a careful consideration of them, with a view of answering the question, What is the measure of utility of lime vapor in such cases?

The first inquiry to this end is, What is the capacity of lime water as a solvent for false membrane? Biermer, in repeating the experiments of Kuchenmeister, observed that membrane placed in lime water in a small glass vessel, disappeared in from ten to fifteen minutes, leaving only a slight sediment. Jacobi observed that lime water required from four to ten hours to liquify soft diphtheric exudations; while, for firm pseudo - membrane, from thirty to seventy - two hours were required. Dr. Meig's found that firm white exudation placed in lime water at a temperature lower than that of the mouth, was reduced to a granular purtrilage in a few hours, the time required for these effects varying with different specimens. In two instances, Dr. DaCosta directed the spray from a solution of lime eight times stronger than the aqua calcis upon diphtheric deposits, for three or four minutes at a time, every few hours, without producing the least impression. In these trials the spray was not hot.

The solution of lime nebulized has varied in strength from that in which the officinal liquor was diluted with thirty parts of water, thus containing one-sixtieth of a grain of lime to the ounce, to that of the liquor calcis saccharatus containing four grains to the ounce.

The use of lime water of these varying strengths is generally quite favorably reported upon. The actual value of the lime, as a curative agent in the cases reported, is impossible of estimation. It has rarely been employed to the exclusion of other means; and whenever used, it has of necessity been in most intimate association with two of the most important remedies for the relief of croup—heat and moisture. Considering the great value of steam in this affection, coupled with the statement of several experimenters that the lime water inhalations were more efficient when the water nebulized was boiling, so as to furnish the spray as hot as possible, and regarding, at the same time, the chemical fact that the hotter the spray the less lime it contains; and bearing in mind the extremely attenuated solutions praised as efficacious, containing, some of them, only one-sixtieth of a grain of the alkali to the ounce, we may well doubt whether lime, as an element of lime water, has been shown to have any efficacy in pseudo-membranous croup.

In March, 1866, Dr. Geiger, of Dayton, Ohio, recommended the following plan for bringing this substance into contact with false membrane in the air passages. He says: "I placed some unslacked lime in a saucer, and after throwing a cloth over the patient's head, I held the saucer under, so that the patient was compelled to breath the fumes arising from the process of slacking. I retained it there for a few minutes." Following Dr. Geiger's successful use of the remedy in this manner, a number of favorable cases have been reported. I regard this mode of using the agent as a vast improvement upon the ordinary plan by nebulization; and the impression that the vapor of lime is being applied in a very imperfect way, by many practitioners now giving it a trial, has emboldened me to come before this Society with my own limited experience with it.

Before proceeding to state the manner and measure in which I now use the lime vapor, I should state that I am indebted to Dr. D. B. Hillis, of Keokuk, Iowa, with whom I have, on several occasions, been associated in the management of cases of membranous croup, for anything of value in the plan. It will be observed that it differs in nothing material from Dr. Geiger's method, excepting that the dose of lime, if I may so express myself, is much larger than that used by him.

Having formed a small enclosure by covering a clothes-horse with sheets, or by taking advantage of the favorable relation of a door to the corner of a room,so as with bedclothes to close in a suitable space, the preparations proceed as follows : To one side of the tent, on a piece of old carpet is placed a small tub; in it is put a common wooden bucket, one - quarter filled with boiling water; at hand is a supply of unslacked lime, and a kettle of boiling water. The nurse and child, or the child alone, if of such age as to remain without an attendant, take position toward the middle of the enclosure, the face of the patient being turned from the tub; by raising the sheet, several pieces of lime, as large as the fist, are placed in the bucket; after a few minutes the evolution of the vapor begins. The physician, through that fold of sheeting forming the door of the tent, frequently takes a view of the steam within, estimating its density by the sight, taste and smell. It is impossible to indicate the proper degree of this density. I should say it should be somewhat less than that of the cloud of steam escaping from the exhaust pipe of a steam engine. The smell and taste of lime should not be too pronounced. The nurse should be instructed to give notice if the steam or heat oppress her, so as to produce a feeling of faintness, sense of suffocation, or irritation of the air passages. Should the vapor be deemed too dense, its intensity may be diminished by opening the flap of the enclosure, or, if need be, by withdrawing the bucket. The pulse of the patient should be noticed from time to time, in view of the possibility of exhaustion supervening, an event said to have occurred in the practice of some physicians. More lime and hot water may be placed in the bucket, as required. The tub is intended to receive any overflow from the bucket, which, in prolonged cases, will require to be emptied.

The quantity of lime required will be large. The increased freedom with which it is now being used may be illustrated by my own experience. In my first trials with it, when asked by the parent how much would be needed, I answered about a quarter of a peck. In a case treated by Dr. O. P. McDonald, of Keokuk, in association with Dr. Hillis and myself, in 1871, the lime baths were used as required for forty-eight hours, during the last twenty-four of which the infant was within the enclosure more than half the time. The quantity of lime used was estimated by Dr. McDonald to be five bushels. It is important to bear in mind that the amount of vapor given off by lime varies greatly. From some specimens very unsatisfactory results are obtained; a half bucket full of an unsuitable article may not give off vapor enough to keep the tent filled for half an hour. The lime should be fresh and well burned. If not sufficiently burned, it is rocky, and slacks badly; glazed pieces, known as " clinkers," should be rejected.

Dr. Geiger speaks of compelling his patients to breathe the lime atmosphere for a few minutes. They should be placed in the tent as often as more urgent symptoms arise, and be kept there for half an hour, or even an hour and a half, till relief occurs, or until it is apparent that no benefit is resulting from the treatment. No experiments have been undertaken to determine the heat of these baths; I should judge, however, that they commonly reach that temperature recommended by Dr. Sayre for the steam bath, ninety degrees. It is quite possible to make the tent too hot, as well as too full of vapor. In an epidemic of cholera, many years ago, I resorted to the use of lime very much in this way, with the hope of restoring heat to the algid bodies of patients in collapse. In several instances the temperature developed was sufficient to scorch the sheets. As a word of caution, it may be well to state that occasionally lime, in slacking, spatters, particularly when it is just from the kiln, and when the quantity of water used is small. Heated particles may thus be thrown some distance. Such an event occurring when the agent is employed as recommended by Dr. Geiger, might lead to painful results. I once observed the lids and conjunctivæ of an infant become inflamed under the use of the baths, a result, probably, of too strong a vapor, or of too prolonged exposure.

According to Dr. Geiger, the effect of this remedy is to cause the breathing to become more easy, and to induce the expectoration of a large quantity of mucus and phlegm. Dr. Hillis writes: "The evidences of relief were quite distinct, and generally speedy; the breathing was improved; the cough decreased, and an increased secretion and ejection of phlegm from the throat took place.

In a word, the onward march of the disease seemed checked at once, and a real improvement established. It was remarkable to observe how soon, when in the bath, the patient was soothed and quieted into a placid sleep. The boy whom we saw, five years of age, was apparently much frightened at the necessary preparations, and at the boiling and sputtering of the lime. He was choking, coughing, and struggling as if his very life depended on his resistance. Shortly he fell into a quiet sleep, and upon awakening expectorated a large quantity of mucus and half dissolved membrane." In short, the lime bath seems to induce free perspiration, to increase the secretion from the air passages, to promote the expectoration of false membrane, and thus to lead to the general improvement of the patient.

The modus operandi of the agent is uncertain; of course, the simplest theory is that it dissolves the false membrane. Some, as Drs. Meigs and Pepper, refer all benefit from its use to the heated steam evolved. Dr. J. L. Smith suggests that the lime bath may be an improvement on the steam bath, in this, that in the latter, on account of the necessity of keeping the room closed, the air soon becomes charged with exhaled carbonic acid, whereas, in the former the expired acid is speedily destroyed by the vaporized lime. May it be possible correctly to extend this idea of Dr. Smith's. Thus, the dyspnœa is in great part a result of the inability of the respiratory organs to relieve the blood of its carbonic acid. By using air, as in the lime bath, charged with a chemical having a remarkable affinity for this acid, may it not be that the pulmonary interchange of gases is advantageously supplemented?

I have knowledge of four cases of membranous croup treated by lime; of these, two were speedily relieved. In a third, recovery ensued, though the lime baths were abandoned for the potash treatment, when the child, though very near death, was thought to be a little better. In the fourth case, the disease had existed one week before medical treatment was sought; an indifferent article of lime was inefficiently used for a time; death resulted. In the last two cases, relief was afforded by the baths; and although they were finally abandoned in one case, and imperfectly used and neglected in the other, there was, in both instances, reason to question the curative power of the agent. In none of these cases was the lime used to the exclusion of other remedies. So far as observed, however, improvement was in no wise referable to the medication.

This mode of treatment is useful in those cases in which the attendant is uncertain of his diagnosis; in which, while he believes he has to do with a case of simple laryngitis, he fears membranous croup. In such instances, the lime bath relieves the distress of the patient, and tends to quiet the anxiety of the practitioner, seeing that he is treating the apprehended disease with no danger of injury to his patient from the *nimia cura medici.*

